# Fine-Needle Aspirates CYFRA 21-1 is a Useful Tumor Marker for Detecting Axillary Lymph Node Metastasis in Breast Cancer Patients

**DOI:** 10.1371/journal.pone.0057248

**Published:** 2013-02-25

**Authors:** Jung Hyun Yoon, Kyung Hwa Han, Eun-Kyung Kim, Hee Jung Moon, Min Jung Kim, Young Joo Suh, Ji Soo Choi, Byeong-Woo Park

**Affiliations:** 1 Department of Radiology, Research Institute of Radiological Science, Yonsei University College of Medicine, Seoul, Korea; 2 Department of Radiology, CHA Bundang Medical Center, CHA University School of Medicine, Seongnam, Korea; 3 Department of Research Affairs, Yonsei University College of Medicine, Seoul, Korea; 4 Department of Radiology, National Cancer Center, Goyang, Korea; 5 Department of Surgery, Yonsei University College of Medicine, Seoul, Korea; Health Canada, Canada

## Abstract

**Introduction:**

To assess whether the value of CYFRA21-1 in the aspirates of ultrasonography-guided fine-needle aspiration biopsy (US-FNAB) can contribute to improving the performances of US-FNAB in the diagnosis of axillary lymph node (LN) metastasis in breast cancer patients.

**Methods:**

US-FNAB was performed in 156 axillary LNs in 152 breast cancer patients (mean age: 51.4 years, range: 17–92 years). Concentrations of CYFRA21-1 were measured from washouts of the syringe used during US-FNAB. Tumor marker concentrations, US-FNAB, intraoperative sentinel node biopsy (SNB), and surgical pathology results were reviewed and analyzed. For comparison, the values of CEA and CA15-3 were also measured from washouts.

**Results:**

Among the 156 LNs, 75 (48.1%) were benign, and 81 (51.9%) were metastases. Mean concentrations of CYFRA21-1 were significantly higher in metastasis compared to benign LNs (*P*<0.001). US-FNAB combined to CYFRA21-1 showed significantly higher sensitivity, NPV, and accuracy compared to US-FNAB alone (all values *P<*0.05). All diagnostic indices of US-FNAB combined to CYFRA21-1 were significantly higher compared to US-FNAB combined with CEA or CA15-3 (all *P*<0.001). Of the 28 metastatic LNs which showed metastasis on SNB, CYFRA21-1 showed higher positive rate of 75.0% (CEA or CA15-3∶60.7%, *P* = 0.076).

**Conclusion:**

Measuring CYFRA 21-1 concentrations from US-FNAB aspirates improves sensitivity, NPV, and accuracy of US-FNAB alone, and may contribute to reducing up to 75.0% of unnecessary intraoperative SNB. Compared to CEA or CA15-3, CYFRA21-1 shows significantly higher performances when combined to US-FNAB in the preoperative diagnosis of LN metastasis in breast cancer patients.

## Introduction

Presence of metastatic lymph nodes in the axilla is the most important prognostic factor in predicting outcome of patients diagnosed with breast cancer [1]–[5] and preoperative diagnosis of axillary or cervical lymph node (LN) metastasis directly affects the type of surgery or treatment of the patient [6]–[8]. Ultrasound (US)-guided fine-needle aspiration biopsy (US-FNAB) is a widely accepted, easy but accurate diagnostic method that can be used in evaluation of the axillary status and showed sensitivity up to 86.4%, specificity up to 100% in several studies [7], [9]–[13]. Although sentinel node biopsy (SNB) is the method of choice in evaluating axillary status, application of preoperative US-FNAB in breast cancer patients who are expected to undergo surgical treatment can reduce SNB, which can be both time consuming and expensive [7], [14]. The NCCN Guidelines for Breast Cancer recommends US-FNAB for initial breast cancer staging [15], and patients who have positive cytology results on preoperative US-FNAB may do without SNB, but those showing clinically negative LNs still need SNB for confirmation of the axillary status. Also, with the development of surgical techniques and postoperative patient management, administration of neoadjuvant chemotherapy has steadily increased with the purpose for breast conserving surgery, therefore, diagnostic methods that can provide accurate information of the axillary status is urgently required. SNB is a highly accurate method in axillary evaluation [16]–[17], but this is an invasive procedure which itself can bring about surgical complications or delayed onset of neoadjuvant chemotherapy [17]. Using non-surgical diagnostic procedures may be of great clinical benefit with simplifying or reducing aggressive surgery just for diagnostic purposes.

Recently, there had been some investigations using tumor markers during evaluation of axillary LN metastasis [7], [18]–[20]. One of them measured the concentrations of the tumor markers carcinoembryonic antigen (CEA) and breast cancer antigen 15-3 (CA15-3) in axillary LN aspirates, and proved that measurements of these tumor markers may be of additional help in preoperative diagnosis of axillary LN metastasis in breast cancer patients [7]. Other studies used sentinel lymph node analysis using one-step nucleic acid amplification (OSNA) has been proposed for intraoperative staging of breast cancer [18], [21], and in these studies cytokeratin 19 (CK19) was considered as the most appropriate marker due to its broad expression in breast carcinomas [18], [22]. Similarly, serum cytokeratin fragment 21-1 (CYFRA 21-1), which is known to react specifically with CK19 fragments, was recently proposed as an independent indicator of prognosis in breast cancer [23]–[24]. Based on the concepts of these studies, we investigated whether the CYFRA 21-1 concentrations of US-FNAB aspirates can contribute to improving the performances of US-FNAB in the diagnosis of axillary lymph node metastasis in breast cancer patients.

## Materials and Methods

### Patients

The Institutional Review Board (IRB) of Severance Hospital, Yonsei University approved of this study, and written informed consent was obtained from the patients included.

**Figure 1:**
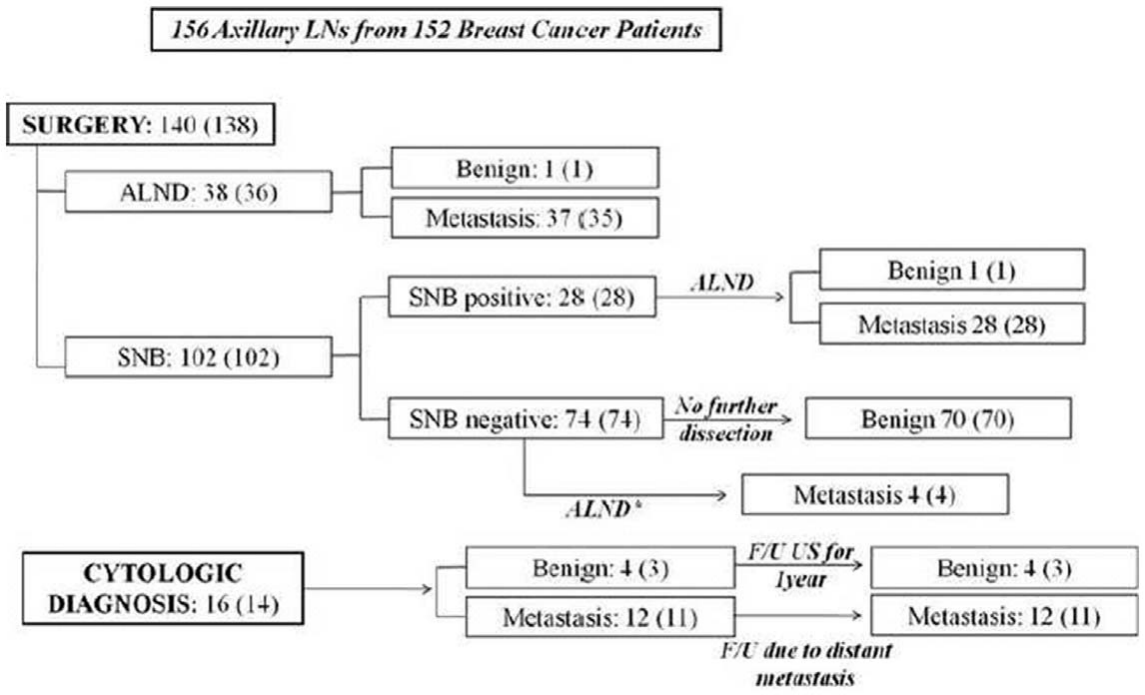
Diagram of diagnosis, surgical management and pathologic diagnosis of the axillary LNs in 152 patients with breast cancer. Note: patient numbers are in parentheses ALND: axillary lymph node dissection. ^*^: ALND performed due to metastatic results on preoperative US-FNAB cytology, All 4 patients had received neoadjuvant chemotherapy, and final pathology showed micrometastatic foci within the lymph nodes.

From March 2008 to July 2010, 152 consecutive women diagnosed with breast cancer who had undergone US-FNAB in 156 axillary LNs at our institution were included in this study (Fig. 1). One-hundred thirty-eight (90.8%) patients had surgery and subsequent SNB or axillary dissection, and the remaining 14 (9.2%) patients were diagnosed based on cytologic results of US-FNAB. These 14 patients had not been operated on due to presence of distant metastasis on other imaging studies (n = 11) or benign cytology results in post-mastectomy patients who had improved or no changes on follow-up imaging studies for at least 1 year (n = 3). Mean age of the patients were 51.4 years, ranging from 17 to 92 years. Primary breast cancers diagnosed with biopsy or surgery are as follows: invasive ductal carcinoma (n = 128), ductal carcinoma *in situ* (n = 13), papillary carcinoma (n = 5), invasive lobular carcinoma (n = 4), mucinous carcinoma (n = 1), and tubular carcinoma (n = 1).

### US and US-guided FNAB

US was performed by one of the seven board-certified radiologists specializing in breast imaging (E.K.K, M.J.K., H.J.M., J.H.Y., and J.S.C), each of whom had various experiences in breast imaging and biopsy (range: 1–11 years), using a 5-12-MHz (HDI 5000; Philips-ATL, Bothell, WA) or 5-12-MHz (iU22; Philips Medical Systems) linear array transducer. Suspicious US features suggestive of metastasis in the axillary LNs were as follows; loss of fatty hilum, cortical thickening measuring more than 3 mm in thickness, irregular or round shape, extracapsular tumor extension, markedly hypoechogenicity of the cortex, and prominent peripheral blood flow on US Doppler [3], [7]. Size was measured as the maximum diameters on either transverse or longitudinal scans.

US-FNAB was performed with a 23-gauge needle attached to a 2-mL disposable syringe using freehand technique. Each LN was aspirated at least twice. Material aspirated were immediately expelled onto glass slides and placed in 95% alcohol for Papanicolaou staining. The remaining contents in the needle and syringe from the first aspiration were rinsed with 1 mL of saline. Concentrations of CEA, CA15-3 and CYFRA 21-1 were measured individually from these washouts. Washouts from the second aspiration were sent for cell block processing.

Five cytopathologists (with 1–15 years of experience) interpreted the FNAB specimen. The cytologic results were divided into two categories based on the reports. Cytologic results showing metastasis from breast cancer or atypical cells were considered positive. Reactive hyperplasia or benign lymphadenopathy, results that are non-related to breast cancer were considered negative. Reports showing insufficient material for specific diagnosis were considered negative, as proposed in other reports [7], [25]–[27].

### Tumor Marker Analysis

Tumor marker concentrations were analyzed with an automated immunoanalyzer system with chemiluminescent immunoassays for CEA (Unicel DXi800; Beckman Coulter, Inc, USA), CA 15-3 (VITROS 3600; Ortho-Clinical Diagnostics, USA), and CYFRA 21-1 (E411; Roche Diagnostics, Germany). A noncompetitive immunometric sandwich assay format containing monoclonal antibodies for each tumor marker was used with the immunoassay reagents (AccessCEA for CEA, Vitros CA 15-3 for CA 15-3, CYFRA 21-1 for CYFRA 21-1).

### Surgical Procedures for Diagnosis of Axillary Lymph Node Metastasis

Figure 1 shows the flow diagram for diagnosis, surgical management, and pathology results of axillary LN metastasis in the patients included in our study. Breast surgery including evaluation of the axillary nodal status was performed by one of the two breast surgeons with 16 and 9 years of experience in breast surgery, respectively. Axillary LNs with positive cytology on US-FNAB directly underwent axillary node dissection, without sentinel node biopsy (SNB). Axillary LNs with negative cytology on US-FNAB underwent SNB, and according to the SNB results, axillary node dissection was performed on patients showing positive histology on SNB, while no further nodal dissection was done if SNB showed negative histology, with the exception of patients with neoadjuvant chemotherapy [7].

### Data and Statistical Analysis

Histopathologic results from subsequent surgery or clinical evidence during follow-up were regarded as standard reference. Among the LNs with surgical confirmation, aspirated LNs were correlated by the location seen during surgery.

Student’s *t*-test was used in comparison of mean size between benign and metastatic LNs. Shapiro-Wilk test and Wilcoxon rank-sum test was used in comparison of the concentrations of each tumor marker between benign and metastatic groups.

Sensitivity, specificity, positive predictive value (PPV), negative predictive value (NPV), and accuracy were assessed based on standard reference for US-FNAB alone, US-FNAB combined with CYFRA 21-1, and US-FNAB combined with CEA or CA15-3. Logistic regression method was used in estimation of cutoff concentration levels of tumor markers. Generalized estimating equation (GEE) method or weighted least square (WLS) method was used was used in comparison of diagnostic performances between US-FNAB alone and US-FNAB combined with CYFRA 21-1, and US-FNAB combined with CYFRA 21-1 and US-FNAB combined with CEA or CA15-3. Fisher’s exact test was used in comparing performances among tumor markers in LNs which had undergone intraoperative SNB.

Statistical analyses were performed with SAS, version 9.1.3 for Windows (SAS Institute, Cary, NC). *P*<0.05 was considered to indicate statistical significance.

## Results

Of the 156 axillary LNs in the 152 patients included, 75 (48.1%) were benign, and 81 (51.9%) were metastases. Mean size of the 156 axillary LNs was 13.07±6.38 mm, ranging from 1.00 to 35.00 mm. Among the positive LNs, 69 were diagnosed with surgery, while the remaining 12 were diagnosed with cytology alone, due to the presence of distant metastasis (n = 11) or plans for chemotherapy or radiation therapy than surgery (n = 1).

**Figure 2:**
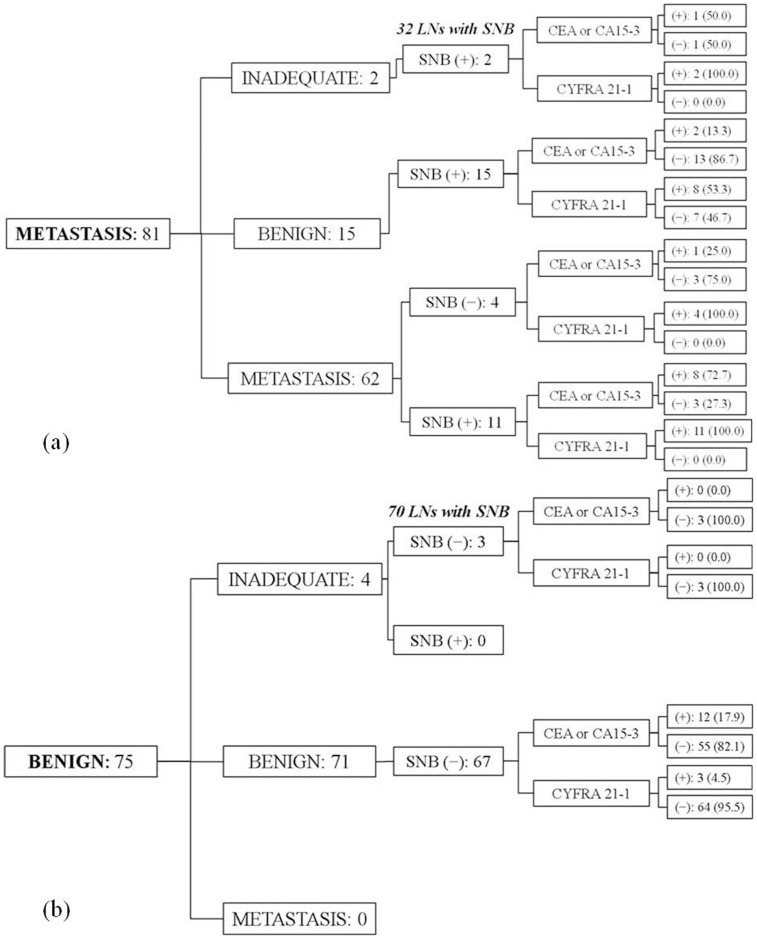
Diagram of US-FNAB, SNB and tumor markers in proven metastatic (a) and benign (b) lymph nodes.

Of the 69 metastatic LNs diagnosed with surgery, 32 (49.2%) underwent SNB prior to axillary LN dissection, among which 28 showed metastatic results (Fig. 2a). Of the 75 benign LNs, 70 (93.3%) underwent SNB, all showing benign results (Fig. 2b). Among the 75 benign LNs, 4 were diagnosed as benign based on cytology only. All 4 LNs in 3 patients were followed regularly with US for at least 1 year, and showed decreased size or no changes on follow-up images.

### Comparison of Size and Concentrations of Tumor Markers

Table 1 shows the comparison of mean size and mean concentrations of the tumor markers between the benign or metastatic LNs. Mean size of the LNs was significantly larger in metastasis compared to benign, 14.43±7.34 mm to 11.60±4.78 mm (*P* = 0.001). Mean concentrations of CEA, CA15-3 and CYFRA 21-1 showed significantly higher levels in metastatic LNs compared to the benign LNs (all markers *P*<0.001, respectively).

**Table 1:** Mean size and concentrations of CA125, CA19-9, and CYFRA 21-1 in US-FNAB aspirates of the 156 axillary lymph nodes.

		Pathology		*P*
		Benign (n = 75)	Malignant (n = 81)	
**Mean LN Size** (mm)		11.60±4.78	14.43±7.34	0.001
**CEA** (ng/mL)	Mean ± SD	0.12±0.16	26.14±101.27	<0.001
	Median	0.03	0.31	
	Min/Max	0.00/0.65	0.00/745.29	
**CA15-3** (IU/mL)	Mean ± SD	2.23±2.00	20.87±87.20	<0.001
	Median	1.80	3.30	
	Min/Max	0.50/17.10	0.50/729.0	
**CYFRA21.1** (ng/mL)	Mean ± SD	1.18±0.75	815.30±1375.17	<0.001
	Median	0.98	94.31	
	Min/Max	0.47/6.32	0.69/4923.0	

### Comparison of Diagnostic Performances of US-FNAB Combined with CYFRA 21-1 and US-FNAB Alone, US-FNAB Combined with CEA or CA15-3

Cytology results from US-FNAB in the 156 LNs included are as follows: inadequate or insufficient material for diagnosis in 6 (3.9%), benign in 86 (55.1%), and metastasis in 64 (41.0%).

Diagnostic performances of CEA, CA15-3 and CYFRA 21-1 alone, US-FNAB, US-FNAB combined with CYFRA 21-1, and US-FNAB combined with CEA or CA15-3 are summarized in Table 2. Sensitivity, specificity, PPV, NPV, and accuracy of US-FNAB alone was 79.0%, 100.0%, 100.0%, 81.5%, and 89.1%, respectively. Among the LNs proven as metastasis on surgery, 17 were diagnosed as inadequate or benign on US-FNAB, showing false-negative rate of 21.0% (17/81).

**Table 2:** Diagnostic performances of CEA, CA15-3 and CYFRA 21-1 at cutoff levels, US-FNAB alone, and US-FNAB combined with each tumor marker.

	Cutoff level	Sensitivity	*P*	Specificity	*P*	PPV	*P*	NPV	*P*	Accuracy	*P*
CEA	0.58 ng/mL)	43.2 (35/81)	–	98.7 (74/75)	–	97.2 (35/36)	–	61.7 (74/120)	–	69.9 (109/156)	–
CA15-3	2.8 (IU/mL)	59.3 (48/81)	–	82.7 (62/75)	–	78.7 (48/61)	–	65.3 (62/95)	–	70.5 (110/156)	–
CYFRA 21-1	1.93(ng/mL)	87.7 (71/81)	–	96.0 (72/75)	–	96.0 (71/74)	–	87.8 (72/82)	–	91.7 (143/156)	–
US-FNAB	–	79.0 (64/81)	–	100.0 (75/75)	–	100.0 (64/64)	–	81.5 (75/92)	–	89.1 (139/156)	–
US-FNAB & CYFRA 21-1	–	91.4 (74/81)	0.001†	96.0 (72/75)	0.085†	96.1 (74/77)	0.002†	91.1 (72/79)	<0.001†	93.6 (146/156)	0.001†
US-FNAB & CEA or CA15-3	–	86.4 (70/81)	<0.001*	81.3 (61/75)	<0.001*	83.3 (70/84)	<0.001*	84.7 (61/72)	<0.001*	84.0 (131/156)	<0.001*

–Raw data in parentheses,

† values compared to US-FNAB,

* values compared to US-FNAB combined with CYFRA 21-1.

Optimal cutoff concentration values of the three tumor markers calculated from logistic regression methods were as follows: CEA 0.58 ng/mL, CA15-3 2.8 IU/mL, and CYFRA 21-1 1.93 ng/mL, respectively. US-FNAB combined to CYFRA 21-1 showed significantly improved sensitivity, NPV and accuracy compared to US-FNAB alone, 91.4% to 79.0% (*P* = 0.001), 91.1% to 81.5% (*P*<0.001), and 93.6% to 89.1% (*P* = 0.001), respectively, but decreased PPV, 96.1% to 100.0% (*P* = 0.002). Specificity of US-FNAB combined to CYFRA 21-1 was lower than US-FNAB alone, 96.0% to 100.0%, but without significance (*P* = 0.085). For comparison with a previous study [7], performances of US-FNAB with CEA or CA15-3 was calculated and compared to US-FNAB with CYFRA 21-1, showing significantly higher values in all diagnostic indices in US-FNAB combined with CYFRA 21-1 (all *P*<0.001).

Figure 2 summarizes the results of US-FNAB, SNB, and concentrations of the three tumor markers. Among the 81 metastatic LNs, 17 had inadequate or benign cytology results on US-FNAB, among which all 17 were diagnosed as positive on SNB. Eight (53.3%) of the 15 LNs showing benign cytology had CYFRA 21-1 concentrations categorized as positive, while only 2 (13.3%) and 3 (20.0%) LNs were categorized as positive when using CEA and CA15-3 concentration levels. Both LNs showing inadequate cytology had CYFRA 21-1 concentrations categorized as positive, while none of the LNs were positive for CEA and one LN was positive for CA15-3.

### Correlation of Sentinel Node Biopsy with Tumor Marker Concentrations

Table 3 shows the correlation of the 102 axillary LNs with SNB to the concentrations of CEA or CA15-3 and CYFRA 21-1. Among the 28 metastatic LNs diagnosed as metastasis on SNB, CYFRA 21-1 showed higher positive rates compared to CEA or CA15-3, 21 (75.0%) to 17 (60.7%), respectively, but without statistical significance (*P* = 0.076). All 4 metastatic LNs diagnosed as benign on SNB showed positive results in CEA or CA15-3 and CYFRA 21-1.

**Table 3:** Correlation of the 102 axillary LNs with sentinel node biopsy to concentrations of CEA, CA15-3 and CYFRA 21.1.

Final	SNB		CEA or CA15-3		CYFRA 21-1	
		N	Negative	Positive	Negative	Positive
Benign	B	70	57(81.4)	13 (18.6)	67 (95.7)	3 (4.3)
70	M	0	–	–	–	–
Metastatic	B	4	0 (0.0)	4 (100.0)	0 (0.0)	4 (100.0)
32	M	28	11 (39.3)	17 (60.7)	7 (25.0)	21 (75.0)

Percentages are in parentheses.

Of the 70 benign LNs diagnosed as benign SNB, CYFRA 21-1 showed significantly higher negative results compared to CEA or CA15-3, 95.7% to 81.4%, respectively (*P* = 0.021).

## Discussion

US-FNAB is a common and popularly used diagnostic method in predicting nodal status of breast cancer patients [4], [6], [9]–[11], [26]. Although not as confirmative as surgical pathology, with fine-needle aspiration surgeons can predict in detail what to expect on surgical pathology based on the cytology results. But, US-FNAB has its limitations in that false-negative results can exist [1], and therefore, patients who are diagnosed as negative or non-diagnostic on cytology alone need additional sentinel node biopsy procedures during surgery to decide upon axillary node dissection, which is both time consuming and expensive. Also, performing axillary dissection on all patients with breast cancer can lead to increased morbidity from various surgical complications. Various efforts have been made to improve the performances of preoperative US-FNAB [1], [7], [9], including measurement of tumor markers used in the management of breast cancer patients such as CEA and CA 15-3 from US-FNAB aspirates [7].

In addition to CEA and CA15-3, CYFRA 21-1 (derived from the words *cy*tokeratin *fra*gment 21.1), the most sensitive tumor marker used in monitoring non-small cell lung cancer [28]-[29], has been reported to be detected in the peripheral blood from patients with metastatic breast cancer and proposed to be used in monitoring disease relapse and treatment response in breast cancer patients [23]–[24], [30]. Also, studies analyzing CK19 in the intraoperative diagnosis of metastatic axillary LNs in breast cancer patients using molecular diagnostic devices [31]–[32], or rapid molecular-based assay during intraoperative SNB [18], [33]–[34], showed high concordance rate to histopathology reducing delayed secondary surgical procedures. Since US-FNAB cytology is an excellent diagnostic tool in the preoperative diagnosis of axillary LNs in breast cancer patients [6], [8], [11]–[13], adding tumor marker analysis to the washouts obtained from US-FNAB procedures has its strong points in that more information can be obtained by this method without addition invasive procedures, in short, both cytology and tumor marker status can be obtained with a single US-FNAB session. Based on this, we investigated the role of CYFRA 21-1 concentrations from US-FNAB aspirates in the diagnosis of metastatic axillary LNs, adapting the concept of measuring the CEA and CA15-3 concentrations from FNAB aspirates of axillary LNs from a previous study [7].

At the present date, administration of neoadjuvant chemotherapy is increasing rapidly with the expectation of breast conserving surgery, even in patients with large breast masses [35]. Acknowledgement of the axillary nodal status is critical in both management and prognosis of these patients [14], and some studies recommend invasive SNB prior to neoadjuvant chemotherapy for more accurate evaluation of axillary status [16]–[17]. Claiming that SNB should be performed prior to neoadjuvant chemotherapy may be due to the false-negative and inadequate results of US-FNAB, a major limitation of this procedure [7], [9], [14], [27], [36]. Our results show that combining CYFRA 21-1 concentrations to US-FNAB can overcome this limitation somewhat; according to the results of our study, US-FNAB combined to CYFRA 21-1 can accurately diagnose axillary LN metastasis with high sensitivity of 91.4%, significantly higher than that of US-FNAB alone, 79.0%. Adding CYFRA 21-1 to US-FNAB significantly improves the sensitivity of US-FNAB, and may prove to be beneficial in the management of 1) patients who are scheduled for surgery in means of reducing SNB, and 2) patients who need histopathologic confirmation of axillary nodal status prior to neoadjuvant chemotherapy, especially without applying additional invasive surgical procedures such as SNB for diagnosis. In addition, for comparison to a previous study using CEA and CA15-3 in the diagnosis of metastatic axillary LNs [7], we calculated the diagnostic performances of US-FNAB combined with CEA or CA15-3. When comparing US-FNAB combined with CYFRA 21-1 to US-FNAB with CEA or CA15-3, all diagnostic indices were significantly higher in CYFRA 21-1. These results further support the efficacy of using CYFRA 21-1 in the diagnosis of metastatic axillary LNs over CEA or CA15-3, being able to get better performances with the analysis of one tumor marker compared to the combined usage of two. Also, as reported in a study on comparing molecular analysis and histopathology for axillary LN staging in primary breast cancer when compared to molecular detection using OSNA assay [37], histopathology of single tissue sections significantly underestimated the frequency of axillary LN metastasis. In contrast, when using OSNA assay, all the metastatic LNs which were underestimated on single tissue sections were diagnosed as metastasis. If cytology specimen are obtained from various areas of the targeted axillary LN during US-FNAB procedures by changing angles of the needle after skin penetration, even if gross cytologic detection of the metastatic cell itself is difficult, CYFRA 21-1 concentrations may prove otherwise. CYFRA 21-1 may not only improve the diagnostic performances of US-FNAB, but may have a role in enhancing the performances of less experienced performers who are more likely to have insufficient or ambiguous cytology results.

False-negative results and inadequate sampling of US-FNAB arouses confusion and frustration to clinicians and patients [7], [9], [14], [27], [36]. When applying tumor marker concentrations, 8 of 15 metastatic LNs with benign cytology showed elevated concentrations of CYFRA 21.1, while 2 and 3 LNs showed elevated concentrations in CEA and CA15-3. Also, of the 2 metastatic LNs with inadequate cytology, both LNs had elevated concentrations of CYFRA 21-1, while none of them had elevated levels of CEA and one had elevated CA15-3 concentration levels. This shows the relative sensitivity of CYFRA 21-1 in detecting metastatic LNs compared to US-FNAB alone and CEA or CA15-3. In addition, we evaluated the diagnostic performances of tumor markers combined to US-FNAB among lesions with intraoperative SNB to see whether tumor markers help in reducing SNB procedures during surgery. Of the 28 metastatic LNs with metastatic results on SNB, CYFRA 21-1 showed higher positive results compared to CEA or CA15-3, 75.0% to 60.7%, respectively. When using CYFRA 21-1 concentrations, nearly 75% patients would be saved from unnecessary SNB, and would able to proceed directly into axillary node dissection. Also, CYFRA 21-1 showed significantly higher negative results compared to CEA or CA15-3 among the proven-benign LNs with benign SNB results, 95.7% to 81.4%, implying less false-positive results among benign LNs.

This study has several limitations. First, a limited number of axillary LNs were included in this study. Second, whether elevated levels of CYFRA 21-1 have effect on the patient’s outcome or prognosis has not yet been verified. Prospective studies with long-term evaluation are anticipated in the future. Third, since this study was conducted on a clinical basis, all patients included were diagnosed with breast cancer, and our results were not validated by a negative control group, i.e., those who were not diagnosed with breast cancer. In a recent study, concentrations of CEA, CA15-3, and CYFRA 21-1 in US-FNAB aspirates among the non-metastatic LNs ranged from 0.02–1.90 (ng/mL or U/mL) [38], values which may substitute the negative control group of our study.

In conclusion, measuring CYFRA 21-1 concentrations from US-FNAB aspirates improves sensitivity, PPV, NPV, and accuracy of US-FNAB alone, and may contribute to reducing up to 75.0% of unnecessary intraoperative SNB. Compared to CEA or CA15-3, CYFRA 21-1 shows significantly higher performances when combined to US-FNAB in the preoperative diagnosis of LN metastasis in breast cancer patients.
